# Why are dreams interesting for philosophers? The example of minimal phenomenal selfhood, plus an agenda for future research^1^

**DOI:** 10.3389/fpsyg.2013.00746

**Published:** 2013-10-31

**Authors:** Thomas Metzinger

**Affiliations:** ^1^Philosophisches Seminar, Johannes Gutenberg-UniversitätMainz, Germany; ^2^Frankfurt Institute for Advanced StudiesFrankfurt am Main, Germany

**Keywords:** consciousness, self-consciousness, minimal phenomenal selfhood, full-body illusions, out-of-body experiences, bodiless dreams, first-person perspective, mind wandering

## Abstract

This metatheoretical paper develops a list of new research targets by exploring particularly promising interdisciplinary contact points between empirical dream research and philosophy of mind. The central example is the MPS-problem. It is constituted by the epistemic goal of conceptually isolating and empirically grounding the phenomenal property of “minimal phenomenal selfhood,” which refers to the simplest form of self-consciousness. In order to precisely describe MPS, one must focus on those conditions that are not only causally enabling, but strictly necessary to bring it into existence. This contribution argues that research on bodiless dreams, asomatic out-of-body experiences, and full-body illusions has the potential to make decisive future contributions. Further items on the proposed list of novel research targets include differentiating the concept of a “first-person perspective” on the subcognitive level; investigating relevant phenomenological and neurofunctional commonalities between mind-wandering and dreaming; comparing the functional depth of embodiment across dream and wake states; and demonstrating that the conceptual consequences of cognitive corruption and systematic rationality deficits in the dream state are much more serious for philosophical epistemology (and, perhaps, the methodology of dream research itself) than commonly assumed. The paper closes by specifying a list of potentially innovative research goals that could serve to establish a stronger connection between dream research and philosophy of mind.

## The relevance of dream research for philosophy of mind—and *vice versa*

This paper has two parts. First, I will develop an answer to the problem of how to isolate the property of “minimal phenomenal selfhood” (MPS; cf. Blanke and Metzinger, 2009), that is, the *simplest* form of self-consciousness. Part 1 is also a case study, presenting a specific example to make a more general point. I want to show how dream research can make a decisive contribution to the philosophical project of conceptually describing the deepest and functionally fundamental layers of human self-consciousness, and in a way that can turn them into proper explananda for empirical research programs. Part 2 will formulate a short research agenda for future cooperation between philosophy of mind and empirical dream research. My goal in this second part is to develop a short, non-exclusive list of particularly promising contact points, in order to demonstrate why and where exactly a much denser cooperation between the two fields would accelerate progress on both sides. From this discussion I will extract a catalogue of desiderata, i.e., a list of the most promising targets for future research.

How do we acquire knowledge about the world? Is it possible to demonstrate the existence of a mind-independent, external world beyond the contents of subjective experience? For centuries, philosophers have debated the reliability of sensory experience, searching for criteria allowing us to distinguish between waking and dreaming, and confronted the challenge of dream scepticism: Could all of this be a dream, including all of my empirical and sensory-based knowledge? As we might in principle be deceived not merely about external reality, but also about our own minds, dreaming not only poses questions for epistemology, but also for the philosophy of mind and consciousness. A central methodological concern shared by philosophers and empirical psychologists alike is the status of first-person reports, for example as gathered from sleep laboratories (cf. Windt, 2013): How reliable are they, and are there really “first-person data” in a literal sense that could be taken at face value, directly entering the process of scientific theory formation? During the last three decades, research on the problem of conscious experience has emerged as a field of systematic, rigorous research in its own right (Metzinger, 1995, 2000; Seth, 2007). Here, dreaming is an important contrast condition for theories of waking consciousness and perhaps it could even serve as a global model of conscious experience in the future, as it presents us with a second global state of consciousness aside from wakefulness (Revonsuo, 2006, 2010). A slightly more modest approach would be a contrastive analysis (Windt and Noreika, 2011), which only focuses on specific aspects of oneiric phenomenology, comparing them to ordinary, pathological, or certain altered states of waking consciousness without yet assuming a complete theory of dream consciousness. Today, the phenomenon of dreaming has not only become one of the most interesting objects of study at the interface between philosophy of mind and empirical research programs, but a genuine research tool in itself. One hope is to use dreams as an instrument that guides researchers to a deeper understanding of consciousness, self-consciousness, and subjectivity.

## Part 1

## What is the MPS-problem?

The MPS-problem is one small and well-defined - but potentially decisive - aspect of the problem of consciousness. Its methodological relevance consists in a simple, but important fact: Minimal phenomenal selfhood is the absolutely central and *necessary* starting point for any conceptually systematic and empirically rigorous research program confronting the subjectivity of consciousness. The problem of consciousness is not one problem, but a whole bundle of problems (see Metzinger, 1995, 2000, 2010^2^ for introduction). Some of these problems are metatheoretical, for example:

- What *counts* as an explanation?
- What is the proper conceptual interpretation of psychophysical correlations?
- What is the epistemological status of first-person reports?
- Are there methodological constraints on the use of autophenomenological reports in experimental design?
- (…)

Other problems are empirical, such as:

- What is the minimally sufficient neural correlate of consciousness?
- What are the functional differences characterizing conscious vs. unconscious information processing?
- If the NCC has been isolated, and if we have a satisfactory computational model of its activity, is there something that could be specified as the overarching “computational goal” of conscious experience in individual organisms?
- In an evolutionary context, was there an adaptive function of consciousness, did conscious processing maximize inclusive fitness?
- (…)

There are many more of such sub-problems. They are all important, and together they constitute a cluster of research targets that today we call “the problem of consciousness.” However, at least from a philosophical point of view, there clearly is something like a *core issue*: What exactly is a “first-person perspective” (1PP; Box 1)? What do we mean by saying that consciousness is “subjective”, for example when developing anti-reductionist and anti-naturalist arguments trying to show that an empirical explanation of consciousness is out of reach (for classical examples see Nagel, 1974; Jackson, 1982; Levine, 1983)?

Box 1 **Levels of self-consciousness**

1. **MPS** (*minimal phenomenal selfhood*; Blanke and Metzinger, 2009)
  Paradigmatic autophenomenological report: <**I *am* this, here and now.**>
  Self-identification as *transparent spatiotemporal self-location*:
  - A conscious self-representation that is not experienced *as* a representation.
  - Satisfaction of transparency-constraint leads to the phenomenology of identification.
  - Data from bodiless dreams and asomatic out-of-body experiences show that pure spatiotemporal self-location suffices for a robust sense of self (sections Asomatic OBEs and Bodiless dreams).
  - MPS can be experimentally controlled (e.g., De Ridder et al., 2007; Ehrsson, 2007; Lenggenhager et al., 2007)
  - Self-location in an egocentric frame of reference creates a weak, purely geometrical first-person perspective (1PP; see below), which is a necessary constituent of MPS.
  - Once MPS has been established, it can function as the origin of richer and more complex forms of 1PP
2. **BA** (*bodily agency*)
  Paradigmatic autophenomenological report: <**I am an embodied agent.**>
  Self-identification with an extended body image, plus causal self-control.
  - A spatially extended, conscious body-representation that is not experienced *as* a representation.
  - A holistic representation of the system as an entity that acts physically, for example by changing its position in space; and/or
  - has the *potential* for physical agency.
3. **AA** (*attentional agency*)
  Paradigmatic autophenomenological report: <**I am a self in the act of controlling its attentional focus.**>
  Typically, self-identification with an extended body image, plus attentional self-control.
  - A conscious self-representation as an entity currently controlling mental resource allocation; and/or
  - having the *potential* for controlling subsymbolic mental resource allocation.
  - Data from bodiless dreams and asomatic out-of-body experiences show that pure spatiotemporal self-location can coexist with AA (sections Asomatic OBEs and Bodiless Dreams).
4. **CA** (*cognitive agency*)
  Paradigmatic autophenomenological report: <**I am a thinking self.**>
  Typically, self-identification with an extended body image, plus high-level cognitive self-control.
  - A conscious self-representation as an entity currently controlling high-level cognitive processing, including quasi-symbolic, conceptual, and propositional contents; and/or
  - having the *potential* for controlling high-level cognitive processing, including quasi-symbolic, conceptual, and propositional content.
  - Data from bodiless dreams and asomatic out-of-body experiences show that pure spatiotemporal self-location can coexist with CA (sections Asomatic OBEs and Bodiless Dreams).
5. **EAM** (*epistemic agent model; see section on Bodiless Dreams*)
  Paradigmatic autophenomenological report: <**I am a knowing self**>
  Conscious self-representation of the system as an individual entity capable of epistemic self-control:
  - as currently standing in and/or actively constructing knowledge relations to certain parts of the world; and/or
  - as having the *ability* to actively establish such relations.
  - AA is sufficient for having an EAM, CA is not a necessary condition.
6. **1PP** (*first-person perspective*; Metzinger, 2003a,b, 2006, 2007; Blanke and Metzinger, 2009)
  Paradigmatic autophenomenological report: <**I am directed at the world**>
  Conscious representation of the system as currently being *directed* at the world, either by bodily or by epistemic agency. Comes in many variations, paradigmatic examples are:
  - Being directed at a physical goal-state via ongoing bodily agency (BA).
  - Being directed at a perceptual object via high-level attentional control (AA).
  - Being directed at abstract cognitive content (e.g., concepts or propositions) via cognitive self-control, quasi-symbolic thought, etc. (CA).

I believe that the 1PP can be naturalized, because it is a naturally evolved epistemic modality, a highly specific representational *format* creating an internal mode of presenting knowledge and information in the brain of a conscious organism. In order to understand what a 1PP is, we must understand what a phenomenal self is. And in order to understand what a phenomenal self really is, we must begin by isolating the *minimal* version of our target phenomenon. We must first focus on those conditions that are not only causally enabling, but *strictly necessary* to bring it into existence.

Why is this strategy advisable? Human self-consciousness is an extremely complex target phenomenon: It is socially, historically and culturally embedded; it has personal and subpersonal functional layers; some of its representational contents are non-conceptual, while others are conceptual or propositional; some content layers are phenomenally transparent while others are phenomenally opaque^2^; all of these layers dynamically interact; there are different levels of embodiment and grounding (Metzinger, 2014); and so on. Therefore, I propose to adopt a heuristic principle, the principle of “explanatory parsimony”: At least at the outset, we should minimize the number of qualitative kinds or *types of self-consciousness* that we try to explain scientifically or that, as naturalistic philosophers, we try to isolate conceptually as empirically tractable entities in the first place. Minimal self-consciousness is an experience that does not have proper parts that could themselves count as a kind or type of self-consciousness. “Explanatory parsimony” simply means that in an extremely complex domain, we must begin by focusing on strictly necessary constituents. We must first ask what the *minimal* form of our target phenomenon actually is. And it is at this point that dream research becomes relevant to philosophy of mind.

## Minimality and the phenomenology of identification

What is the simplest form of self-consciousness, both in dreaming, and in waking? What is the minimal “phenomenal self-model” (PSM; Metzinger, 2003a, 2007; Blanke and Metzinger, 2009), or the minimal global system model that allows a given conscious system to experience itself as a single and distinct autonomous entity, as a self (Limanowski and Blankenburg, 2013)? What, functionally speaking, *constitutes* such an internal model? “Single” means that a system represents itself as a unified, countable individual, possessing numerical identity. “Singularity,” defined in terms of “unification” and “indivisibility,” implies that some sort of *integration* has taken place. Therefore, we must look for all those functions in the brain that (a) contribute to global self-representation, and (b) dynamically integrate information and representational contents from more than one source, while (if we also want minimality) (c) none of those contents can themselves be taken to be a representation of the system as a whole. “Distinctness” means that, necessarily, a self/world boundary exists. Normally this will include the additional representation of a structured environment involving other unified, countable individuals. One related phenomenological constraint is that we experience ourselves as *parts* of the world, so we must look for those neurally realized functions that seamlessly embed the self-model into a given multimodal scene or situation. And saying that a system treats itself as an “entity” means that it must have attributed a specific *ontological status* to itself: It experiences itself as existing. However, the phenomenology of existence—of the “realness” of the self—has nothing to do with the self-attribution of some property in a linguistic, conceptual, or cognitive sense. It is a pre-reflexive aspect of subjective experience^3^.

## Minimal phenomenal selfhood: the original hypothesis

In 2009, Blanke and Metzinger introduced the concept of “minimal phenomenal selfhood” (MPS). MPS can be analyzed on phenomenological, representational, and functional levels of description. Central defining features are (1) a globalized form of identification with the body as a whole (as opposed to ownership for body parts), (2) spatiotemporal self-location, and (3) a 1PP. It is important to differentiate between a weak and at least two stronger readings of the term “1PP.” A weak 1PP is a purely geometrical feature of a perceptual or imagined model of reality and has formed the target of empirical studies investigating visuospatial perspective-taking (see, e.g., Pfeiffer et al., 2013). For example, in the human case a unimodal (e.g., visual) weak 1PP typically includes an egocentric spatial frame of reference, plus a global body representation, with a perspective originating *within* this body representation. There is a center of projection that functions as the geometrical origin of the “seeing” organism's passive perspective.

MPS is the central enabling condition for having a subjective, consciously experienced 1PP on the weak reading; it is a necessary (but not sufficient) condition on two stronger readings of the term “1PP”^4^. Having MPS is a necessary condition for the strong (i.e., attentional and/or cognitive) 1PP, but not for the weak 1PP, because the weak 1PP is itself a constituting factor for MPS. The notion of “embodiment” is of central relevance for MPS, as is the question of how critical global bodily properties like self-identification, self-location and 1PP can be grounded and functionally anchored in the brain (Metzinger, 2014). In standard configurations, the conscious body-model defines a volume within a spatial frame of reference (the dimension of “self-location”) within which the geometrical origin of the weak 1PP is also localized. Self-location possesses a variable internal structure: the bodily self is phenomenally represented as inhabiting a *volume* in space, whereas the weak 1PP is projected from an extensionless point, the geometrical origin of our perspectival visual model of reality. Normally this point of origin is embedded *within* the volume defined by bodily self-representation (though it can be dissociated)^5^. Because our conscious body model is transparent, that is, because it cannot introspectively be recognized *as* a model, we fully identify with its representational content and enjoy the phenomenology of presence as real, distinct, unified and individual bodily selves.

### Difficulty 1: indeterminacy of the object of identification

In an important criticism, Adrian Alsmith has argued that a set of classical studies on the experimental induction of “full-body illusions” (FBIs; see Blanke, 2012 for review) are basically a misnomer. His point is that the possibility has not been excluded that they only create a *partial* identification with the avatar used as an alternative, second body-representation in these experiments. Alsmith (né Smith) writes:

Unfortunately, the question of whether or not there *are* full-body illusions is empirically under-determined, as putative full-body illusions are difficult to isolate from illusions involving composite parts that to do constitute a “full” or “whole” body. That is to say, a plausible alternative is that only representations of the body parts directly stimulated become subject to the experimentally induced bias, whilst other parts remain relatively (perhaps even completely) unaffected. (…)

The difficulty is that there is little assurance that this is a manipulation of a global representational process. In particular, the issue arises because there is no measure of the extent to which the effect is localized to particular parts. For claiming that the illusion is a full-body illusion surely involves the assumption that it is *not* localized to particular parts (Smith, 2010, p. 38p).

First, to avoid potential conceptual confusions, let us differentiate the issue of *empirical* under determination of the existence of FBIs and the *phenomenal* indeterminacy of the object of identification. The latter might or might not underlie the first. One important distinction is the one between local and global ownership (Blanke and Metzinger, 2009: 9; Petkova et al., 2011; for a useful discussion see Lenggenhager, 2009, section 4.2.3). For example, in a disorder that has been called somatoparaphrenia, the phenomenology of ownership for a body part may break down following damage to right temporo-parietal cortex (Vallar and Ronchi, 2009). Somatoparaphrenic patients most often misattribute their contralesional hand as belonging to another mostly familiar person such as their doctor, nurse, a hospital neighbor, or friend. Somatoparaphrenia must be distinguished from other bodily symptoms reflecting disturbed partial ownership and/or agency such as xenomelia (the persistent desire for healthy limb amputation, caused by the oppressive feeling that one or more limbs of one's body do not belong to one's self, formerly labeled “body integrity identity disorder” or BIID, McGeoch et al., 2011; Brugger et al., 2013; Hilti et al., 2013; van Dijk et al., 2013), anosognosia for a paralyzed limb, alien hand syndrome, depersonalization disorder, congenital and supernumerary phantom limbs, or selective loss of the visual body-representation (as in asomatoscopia, negative heautoscopy, see Dieguez and Blanke, 2011; Dieguez and Annoni, 2013). If one takes reports of these symptoms seriously and uses them to develop a set of heterophenomenological constraints^6^ for any satisfactory theory underlying human self-consciousness, in particular of the identification component, one immediately begins to see the complexity of our high-dimensional, variable inner landscape of bodily self-consciousness. Alsmith's point about the potential empirical under determination of bodily self-identification has only been empirically demonstrated for local patterns of identification relating to the experience of single fingers during the rubber hand illusion (Tsakiris and Haggard, 2005; Tsakiris et al., 2006, p. 427). But it might even be possible that there is no empirical fact of the matter, as there is a deeper underlying problem, namely, the possibility of phenomenal indeterminacy. This would mean that the distinction between global and local body ownership is not given in phenomenal experience itself and is only an artifact of our way of describing it. However, at the very least, Alsmith's argument certainly implies that relevant phenomenological aspects like “identification” and “ownership” cannot be captured by a simple global/local distinction, but have to be described in their fine-grained variance over time (Longo et al., 2008; Tsakiris, 2010; Longo and Haggard, 2012). And this leads to an interesting new version of our central question: What exactly is the *minimal unit of identification*?

### Difficulty 2: does the distinction between self-identification and self-location collapse?

There are two classes of phenomenal states in which we find a robust, temporally stable form of self-consciousness in the absence of an extended body representation: “asomatic” out-of-body experiences (OBEs) and “bodiless” dreams. These state-classes are highly relevant, because they help us refine the theoretical constraints for a more comprehensive theory of self-consciousness, and, more simply, because they demonstrate a *possibility* that any such theory would have to explain.

#### Asomatic OBEs

Out-of-body experiences are episodes of phenomenal consciousness during which subjects experience themselves as being located outside of their physical bodies, often (but not necessarily) involving a perceptually impossible external perspective from which the physical body is seen, heard, or felt. An “asomatic” OBE is one in which the experiential subject lacks any spatial form, because it is phenomenologically identified with an extensionless point in space, external to the physical body as represented (Green, 1968: 17; Irwin, 1985: 8p; Box 2). “Parasomatic” OBEs are characterized by the subjective experience of being embodied in a spatial volume. This can involve phenomenologically either identifying with a double body, which may share many of the features of the physical body as visually represented, or with an indeterminate form, but again external to the physical body as represented. “Aparasomatic” OBEs are a third phenomenological category, in which some sort of counterpart of the experient's physical body is actually represented alongside the physical body, but in which the subject neither identifies with the double nor the physical body as phenomenally represented. For example, a German study found 18.8 % of subjects describing their OBEs as bodiless, with 34.4% perceiving themselves as “being located in a body resembling my own physical body” and 21.9% found themselves “in a cloud, fog, or at a point in space” (Wolfradt, 2000/2001; English translation TM). Depending on the study (see Alvarado, 2000 for a comprehensive review), at least a third of the subjects find themselves located in a second body (but this may also be an indefinite spatial volume) and, on average, about 31 percent of OBEs are actually asomatic—they are experienced as bodiless and often include an externalized visuospatial perspective only^7^. However, the object of identification can also change in the course of one single conscious episode; for example, an asomatic OBE might gradually be transformed into a parasomatic OBE, etc. Carlos Alvarado writes:

Some experience being in another body, usually resembling their physical one (*n* = 10 studies; *M* = 46%, *Mdn* = 49%; range = 15–75%). Others do not experience a body at all, describing themselves as “pure consciousness” (*n* = 6 studies; *M* = 31%: *Mdn*. = 21.5%; range = 7–80%) or as “balls of light,” “points in space,” or “clouds” (*n* = 6 studies; *M* = 29%; *Mdn* 28%; range = 13–47%). (Alvarado, 2000, p. 186p).

Box 2 **Glossary of Terms.**

**OBE**

An “out-of-body experience,” in which the experiential subject has the feeling of being located outside of its physical body as represented, often in combination with a perceptually impossible external perspective. Asomatic OBEs are characterized by MPS, an EAM, plus a weak spatial 1PP (see Box 1; Metzinger, 2003a, 2009).

**Asomatic OBE**

An out-of-body experience in which the experiential subject lacks any spatial form, because it is phenomenologically identified with an extensionless point in space, external to the physical body as represented. Asomatic OBEs are characterized by MPS, an EAM, plus a weak spatial 1PP (see Box 1).

**Dream**

A complex hallucinatory experience occurring in sleep or during sleep-wake transitions, in which the experiential subject is fully immersed and localized in a spatiotemporal scene. Dreams are characterized by severe, and frequently unnoticed deficits in memory, cognition, and rationality. AA and CA are mostly absent, the EAM is highly unstable (see Box 1; Windt, 2010, 2014).

**Bodiless Dream**

A dream in which conscious body representation is reduced to an extensionless point in space, and there is self-identification plus, typically, a knowing self. Most bodiless dreams seem to be lucid dreams, and as such they are characterized by MPS, an EAM, plus a weak spatial 1PP (see Box 1). In non-lucid bodiless dreams, the EAM is absent or unstable.

**Lucidity**

The rare phenomenon that a dreamer is aware of the fact that he or she is currently dreaming. Lucidity can be conceptually analyzed as the gradual stabilization of an EAM. There are different types and stages of lucidity (Windt and Metzinger, 2007; Noreika et al., 2010), and there has been recent progress in isolating the neural correlates of such transitions (Voss et al., 2009, 2013; Dresler et al., 2012). The “meta-awareness” that is regained in dream lucidity may be interestingly related to the termination of mind-wandering episodes during waking periods (Schooler et al., 2011; Metzinger, under review).

**Mind Wandering**

Episodes of phenomenologically spontaneous, stimulus- and/or task-unrelated thought (daydreaming, autobiographical planning, waking fantasies, depressive rumination, etc.). Conceptually, mind wandering can be described as the absence of CA and AA, and as a transient loss of mental autonomy (M-autonomy; see Metzinger, under review). It is empirically plausible to assume that large parts of the neural correlate of mind wandering overlap with activity in the default mode network (DMN; Buckner et al., 2008; Christoff, 2012; Christoff et al., 2009; Stawarczyk et al., 2011).

**M-Autonomy**

The term “M-autonomy” (for “mental autonomy” as opposed to autonomy in bodily or social action) refers to the specific ability to control one's own mental functions, like attention, episodic memory, planning, concept formation, rational deliberation, or decision making, etc. This ability can be a form of rational self-control, which is based on reasons, beliefs, and conceptual thought, but it does not have to be. The central defining characteristic is the “veto component”: Being mentally autonomous means that all currently ongoing processes can in principle be suspended or terminated. This does not mean that they actually *are* terminated, it just means that the ability exists, and that the person has knowledge of this fact. A frequently recurring *loss* of M-autonomy is one major characteristic of our cognitive phenomenology, and it interestingly characterizes dreaming as well as waking life, whereas lucidity can be seen as M-autonomy during the dream state [see (Metzinger, under review) for discussion and references].

**Phenomenal Unit of Identification**

In most dream and waking states there is a unit of identification (UI), determining the conscious experience of “I *am* this!” In dreams, the UI can be highly variable, a fact that potentially lends itself to a new way of categorizing dreams and constructing a novel taxonomy.

**Minimal UI**

The minimal UI is the *simplest* phenomenal property available for subjective identification. In asomatic OBEs and bodiless dreams the UI is transparent spatiotemporal self-location (see Box 1).

**Maximal UI**

The maximal UI is the most general phenomenal property available for subjective identification. Arguably, this is the experiential quality of consciousness *per se* (i.e., phenomenality as such) or the integrated nature of our global conscious model of reality (the “unity of consciousness”). In some aparasomatic OBEs and during advanced states of meditative practice the experiential subject seems to be identified with the maximal UI.

The most important conceptual implication is that we have not only a plausible candidate for the *minimal* unit of identification (see section Difficulty 1: Indeterminacy of the Object of Identification. above), but also a candidate for what I would like to call the *maximal* unit of identification (Box 2). What is the “maximal unit of identification”? It is the possibility of identifying with the most *general* phenomenal property available for identification at all: Philosophers might call it the global “unity of consciousness,” or phenomenality *per se*, or awareness *as such*, namely the singular, integrated, all-pervading quality of conscious*ness* characterizing the current totality of experiential contents, as it is given in every single moment of experience.

Here, the first general insight is that the identification component of phenomenal self-consciousness can in principle be attached to a whole range of different phenomenal properties. It follows that there must be a neural mechanism underlying the phenomenology of identification. It is also plausible to assume that the *function* implemented by this mechanism is domain-general, because it can operate on a variety of representational contents and/or phenomenal properties. Call this the “UI-theory”: Conscious experience can be described as a trajectory through a high-dimensional state space; in navigating the world and orienting themselves, human beings constantly search for a source of maximal invariance, and in our own case we always *identify* with a given region of maximal invariance. The phenomenal unit of identification (UI) can change dynamically, whenever the system discovers a new region of maximal invariance.

Let us look at some informative examples. A standard phenomenal property serving as the “target” of this hypothetical and as-yet unknown mechanism would be the integrated contents of our current body image, another typical example is the subjective quality of “agency” in the control of bodily actions. Often both target properties coincide and simultaneously function as the locus of identification. This is why, in standard situations, we experience ourselves as embodied agents. *Prima facie* one might think that the phenomenal self simply is wherever there is an experience of causal control, for example in bodily agency. However, the data about asomatic OBEs and bodiless dreams show a more differentiated picture. First, explicit body representation is not necessary, because an extensionless point in space is sufficient for phenomenal self-identification. Second, bodily agency is not a necessary condition either, because during these episodes the experience of mental self-control and epistemic agency suffices for creating a 1PP. Third, as the sense of selfhood seems to remain robust even in those asomatic OBEs and bodiless dreams in which there is neither an experience of motion in space nor goal-directed mental activity, it is plausible to assume that agency is not a constitutive element for the minimal unit of identification.

But what other candidates for the UI are there? A more specific example would be the geometrical origin of the visuospatial 1PP, as an extensionless point in space (Blanke and Metzinger, 2009). And the most *general* target property for the mechanism generating the distinct experience of “I *am* this!” is the unity of consciousness as such. This is, of course, a fact already well known from the phenomenology of meditation and prolonged spiritual practice^8^. Long-term meditators describe states of consciousness in which they identify with the non-personal quality of conscious awareness *as such*. However, it is interesting to note how the “pure consciousness” variant of self-identification can also be found in OBEs and dreams (see section Bodiless Dreams below).

The existing empirical material seems to be ambiguous between asomatic OBEs in terms of the only locus of identification being the arguably *minimal* unit of an extensionless point in space, and aparasomatic OBEs in terms of the “pure consciousness” variant (i.e., the unit of identification being phenomenality *per se*, or the global unity of consciousness *as such*, as frequently described when referring to mystical or meditation experiences, see Fasching, 2008; Hölzel et al., 2011). This is partly a conceptual issue, because it is not clear whether pure, minimal self-location in terms of identifying with an extensionless point in space counts as the minimal form of being embodied or not. It is also unclear exactly which type of experience subjects had in mind when they were asked about “bodiless” experiences^9^. For the 18.8% of subjects describing their OBEs as “bodiless” in the Wolfradt study cited above, it is not clear that some of them did not actually belong to the subset of those 21.9% who found themselves “in a cloud, fog, or *at a point in space*” (emphasis TM). For the classic review of Alvarado cited above, it is not entirely clear how many of the 31% of “pure consciousness”-subjects were perhaps also experiencing themselves as visuospatial “points in space” (a subset of the 28%), because it is not exactly clear what subjects (and investigators) actually understood or meant in the questionnaire. It is plausible to assume that an egocentric model of perceptual space was still in existence in both types of phenomenal configuration—but exactly what was the unit of identification? In aparasomatic OBEs it might well have been the *maximal* unit of “pure” consciousness as such. Many of those subjects having experienced an identification with an extensionless point in space may actually *misdescribe* their experience as “pure consciousness”, while still having had a weak 1PP (as defined in Blanke and Metzinger, 2009).

Here is the philosophical puzzle: What, conceptually, should be the semantic cut-off point between genuine bodily *self*-consciousness and the minimal experience of mere conscious self-location at an extensionless point in space? In analytical geometry, a point is a null-dimensional element of a specific vector space, all other geometric objects can be described as sets of points. On the level of mental representation, a point in space clearly is a form of spatial mental content^10^. Therefore, our theoretical framework could perhaps be made more parsimonious, namely by conceptually reducing minimal self-consciousness to spatiotemporal self-location. But what, phenomenologically, is the difference between “I *am* this, here and now!” and “**This**, here and now!”? This is where dream research becomes important for philosophers of mind: Philosophers of mind can greatly profit from the data and fine-grained phenomenological constraints produced by empirical research programs on dream consciousness, and dream researchers should listen to philosophers in refining their own questionnaires and experimental designs.

#### Bodiless dreams

A rare, but well-known phenomenon are dreams in which there is only an *abstract* self-representation in terms of a model of the dreamer as an epistemic subject, identified with an extensionless point in perceptual space. What exactly does this mean? In a first-order approximation, there seem to be three central defining characteristics:

- Minimized spatial content (body perception is reduced to a point in space);
- Phenomenal epistemicity (the stable experience of being a *knowing* entity);
- Self-identification.

“Abstract self-representation” means that the currently conscious self-model does not contain any perceptual or spatially extended features of bodily content: There is no visual, auditory, olfactory, gustatory, tactile, proprioceptive, vestibular, nociceptive, thermal, or interoceptive information represented on the level of bodily self-consciousness (for an example, see Cicogna and Bosinelli, 2001, p. 32, Ex. 11). Spatial embodiment is minimal, because it is confined to an extensionless point in space (Box 2). “Epistemicity” means that the dreamer is modeled as a *knowing self*, as an entity that has knowledge about the world, but also actively expands this knowledge, for example through controlling the focus of attention or by remembering, forming new concepts, active inference, and so on. Most bodiless dreams occur during lucid dreams, and it only is here where the knowing self is truly stable over time. “Phenomenal epistemicity,” as the second defining characteristic, simply means that on the level of conscious experience, the dream self is represented as something that stands in an epistemic relation to the world, in the relation of knowing, thinking, actively guiding attention, of just trying to understand what is going on. Therefore, let us call this type of conscious self-representation an “epistemic agent model” (EAM)^11^. In bodiless dreams, this abstract EAM is the unit of identification. It generates the phenomenology of “I *am* this abstract, knowing self!” Of course, there can be multiple EAMs in social dreams—other dream characters representing other knowing selves—but only one of them serves as the locus of identification. Please also note that the presence of an EAM as the unit of identification does not imply that any form of knowledge, in a more rigorous epistemological sense, is actually in existence: All we have is the *phenomenology* of knowing. The third major defining characteristic is self-identification, namely, at or with an extensionless point in perceptual space and with the EAM. Bodiless dreams are still perceptual spaces, they have an egocentric geometrical structure including a point of origin and a perspective, and therefore, they automatically include a weak 1PP in the sense of Blanke and Metzinger (2009). However, self-location is also maximally abstract: It only includes a point in space and a point in time, a Here and a Now. Let us look at one simple example:

I was thinking of problems about my examination … I had the image of the open book … nothing else. (Occhionero and Cicogna, 2011, p. 1013; see also Occhionero et al., 2005, p. 79, Example 2)

First, we have a rudimentary perceptual space, containing only one object (the book) and one sensory modality (vision). Interestingly, it is not clear if it is phenomenologically adequate to speak of full immersion or a complete “situation model” here (because the “image of the book” might have been floating in space by itself), but there is a visual observer in an egocentric frame of reference—if there weren't, the experience of seeing of the book would have been described in a very different manner. Second, there is an EAM: There is a cognitive subject, a thinker of thoughts about the upcoming examination, and we can plausibly assume that an attentional agent, a subject of attentionally controlled visual content was also given in the experience.

The most relevant point is that in bodiless dreams the abstract unit of *identification* and the unit of *self-location* coincide: The EAM is represented as identical with the origin of the weak 1PP. Therefore, it may be possible to *integrate* conditions 1 and 2 stated in our original definition of MPS, because recent research has demonstrated the high prevalence of asomatic OBEs and bodiless dreams in which the sense of selfhood remains stable. Conceptually, the self-identification condition would then be reduced to the self-location condition, and we would have arrived at a more parsimonious definition of minimal self-consciousness. As Jennifer Windt (2010, p. 312) has pointed out, a better and refined definition of MPS could then be as “transparent self-location in a spatiotemporal frame of reference.” This generates two interesting new questions for research, one conceptual, one empirical:

- Is the “epistemicity” of the EAM a necessary condition for the occurrence of a robust form of phenomenal selfhood? In other words, is every conscious self necessarily a (phenomenologically) *knowing* self, or should we treat the conjunction of “transparency” and “self-location” in itself as a sufficient condition?
- Can dream research demonstrate the occurrence of dreams in which the second element of Windt's definition is given, but in which the transparency constraint is not satisfied? In other words, are there *fully* lucid, phenomenally opaque, and bodiless dreams for which it is true that spatiotemporal self-location is itself experienced *as a form of (mis)representational content*?

In developing her own definition of dreams as “immersive spatio-temporal hallucinations” (ISTH) Jennifer Windt writes:

I suggest that the crucial factor that distinguishes dreaming from non-dreaming sleep experiences is precisely the sense of spatial and temporal presence in the dream. In a very basic sense, there is a hallucinatory scene that is organized around an internal, spatiotemporal first-person perspective (1PP) as well as a sense of spatiotemporal self-location, i.e., the sense of occupying a space (even a point will be extended in a minimal sense), plus an experienced “now” and the experience of duration. While Blanke and Metzinger (2009) use the term weak 1PP to refer to a visuospatial or auditory 1PP, I use it here in the more reduced sense of a purely spatiotemporal 1PP. On this level, I think the distinction between a spatiotemporal 1PP and a sense of spatiotemporal self-location disappears: both refer to the phenomenological property of being located at (and relative to) a certain point in space at a certain point in time. (2010, p. 304)

And she goes on to explain:

Finally, if this analysis of dreams as ISTH is correct, it shows that a minimal form of self-experience or minimal phenomenal selfhood (MPS) does not require what Blanke and Metzinger (2009: 12) call “a passive, multisensory and globalized experience of ‘owning’ a body.” They suggest that this sense of “‘global ownership’—functionally defined as availability of an integrated, transparent, and global representation of the spatiotemporally situated body—is the simplest form of self-consciousness.” While I agree with their conclusion that MPS “is constituted by something ‘less’ than agency” (Blanke and Metzinger, 2009: 12), I would submit that the subjective sense of presence—or at least the retrospective description of an experience as having involved the presence of a self—involves something even less: the sense of immersion or of (unstable) location in a spatiotemporal frame of reference. Neither global ownership nor a visual 1PP, however, are necessary for MPS. For this reason, the ISTH model of dreaming is also directly relevant for the philosophical understanding of self-consciousness. (ibid., p. 306).

Now we can state more clearly why dream research is relevant for philosophers: It is one of the best currently available scientific strategies for grounding the 1PP (see Barsalou, 2008; Metzinger, 2014 for the relationship between “grounded cognition” research programs and subjectivity). It is also the best global contrast condition for isolating MPS. There are only two other sources for investigating MPS and bodiless subjectivity, i.e., states in which body representation is absolutely minimal, but in which a stable sense of selfhood and an “asomatic 1PP” can be found: The scientific observation of OBEs and meditation research. However, both asomatic OBEs and “pure consciousness” experiences in meditators are rare phenomena. The prevalence of spontaneously occurring and asomatic OBEs in healthy subjects is simply too low to make them amenable for investigation under controlled conditions in sleep labs (see Irwin, 1985; Blackmore, 1982/1992 for substantial overviews). The second wave of rigorous meditation research has only begun (see Lutz et al., 2007; Chiesa and Serretti, 2010; Hölzel et al., 2011 for review), but it seems clear that states of consciousness in which subjects identify with the non-personal quality of conscious awareness *as such* only occur in few long-term meditators or trained monks, and often unpredictably. In addition, autophenomenological reports from OBEers and long-term adherents to a specific form of spiritual practice may also be characterized by stronger ideological biases and distortions through individual belief systems than dreams. There is considerable promise in virtual reality (VR) as new tool for research (see Sanchez-Vives and Slater, 2005; Slater et al., 2010) in this area, but as yet all such novel experimental strategies are heavily constrained by the fact that they take waking consciousness and its interoceptively and perceptually rich self-model as an inevitable starting point (Seth, 2013). Dreaming occurs spontaneously in 7 billion people on the planet, and multiple times every night. Thousands of dream reports gathered by scientific researchers are already in existence, and they could be statistically re-evaluated. Bodiless dreams are probably rare, but as dreams are generally more frequent and easier to evaluate, there is still a better chance of finding such reports than for OBEs. Although many (but not all) reports of “asomatic dreaming” seem to come from lucid dreams (LaBerge and DeGracia, 2000), thereby making the target phenomenon potentially more controllable, there is also an inherent risk of specific biases in this subset. Most importantly, the *questions* asked by dream researchers should be refined, for example in accordance with our discussion above, resolving conceptual ambiguities inherent in existing studies. Here is a non-exhaustive list of desiderata for future research:

- What is the minimal unit of identification in the dream state?
- What is the maximal unit of identification in the dream state?
- Are there “minimax” states, in which a subject, for example, feels identical with an extensionless point in space *and* the unity of consciousness at the same time?
- Are there opaque types of spatiotemporal self-location (i.e., without the accompanying phenomenology of self-identification), for example during “aparasomatic” lucid dreams? What is their prevalence?
- In the absence of an EAM, is there any empirical evidence for other forms of conscious self-representation in the dream state?

## Part 2

## Philosophy and dream research: promising contact points

The central claim of this paper is that empirical research on dreaming is highly relevant for philosophy of mind and cognitive science, as well as for the flourishing interdisciplinary field of consciousness research in general. It shatters theoretical intuitions, offers a wide range of important bottom-up constraints for a comprehensive theory of mind, and thus is a constant provocation for old-school analytical epistemology and armchair philosophy of mind. But modern philosophy has a lot to offer to dream researchers as well: More precise phenomenological descriptions, thorough conceptual clarification, and methodological criticism. Let us look at four short examples where interdisciplinary cooperation could be most innovative and promising.

### Example 1: the concept of a “first-person perspective”

For philosophers, the concept of a “first-person perspective” is highly relevant, and for a wide range of reasons. For example, one classical philosophical issue is the question of what exactly it means that conscious mental states are “subjective” states. What exactly does it mean that conscious experience is often bound to an individual 1PP? We lack an empirically grounded theory of subjectivity, a model of the 1PP as a naturally evolved phenomenon. Here, my point is that having a 1PP is not a unitary, but a graded phenomenon, and that dream research can make decisive contributions in functionally dissociating different levels of this specific research target.

An important conceptual distinction is between two ways of being directed at the world as a knowing self, one, *by attention*, and two, *by cognition*, that is, either by a subsymbolic, non-linguistic process of selective precision optimization and resource allocation (i.e., on the level of perception) or by being a subject that is directed at the world by forming new concepts about it. There clearly is a phenomenological difference between being an attending self and being a thinking self. And on a functional level it is, for example, plausible to assume that there are many animals on this planet that can selectively control their attention, but are unable to form concepts. A stronger contrast exists between human non-lucid dreams and human standard wake states, where in the dream state both types of 1PP are either absent or extremely unstable and short-lived, as opposed to the more robust and temporally extended subjective perspective of waking life. In particular, the transition toward a richer and more stable 1PP can be precisely observed in the different stages of prelucidity and lucidity. Because this contrast is stronger during dreams than in wakefulness, the non-unitary and graded nature of the 1PP can be investigated in a much clearer way in dreams than in waking consciousness.

Above, I introduced the concept of an “epistemic agent model” (EAM), as a specific type of conscious self-representation. This means that on the level of conscious experience, the self is represented as something that stands in an epistemic relation to the world, in the relation of knowing, thinking, actively guiding attention, of just trying to understand what is going on. Dream research could allow us to differentiate the functional and representational layers that constitute an EAM. To see the contact points between philosophy and dream research more clearly, let us look at the very specific connection between subjectivity and attention in the dream state.

Attentional agency (AA; see Metzinger, 2003a: 6.4.3., 2006; Box 1) is a phenomenal property, as is the case for pain or the subjective quality of blue in a visual color experience. AA is the conscious experience of initiating a shift of attention, of controlling and fixing its focus on a certain aspect of reality. We know that this property is selectively missing during non-lucid dreaming, but likely also during infancy, dementia, or severe intoxication syndromes. AA involves a sense of effort, and it is the phenomenal signature of the functional ability to actively influence what you will know, and what, for now, you will ignore. AA is fully transparent: The content of your conscious experience is not one of self-representation or of an ongoing process of self-modeling, of depicting yourself as a causal agent in certain shifts of “zoom factor,” “resolving power,” or “resource allocation,” and so on, but simply of *yourself* selecting a new object for attention.

Under a predictive coding approach (Friston, 2010), having an attentional 1PP can be fruitfully associated with second-order statistics or precision optimization (Hohwy, 2013) and with the internal modeling of mental resource allocation (Metzinger, 2003a), but on the functional level of *global availability* (i.e., consciously) and on the representational level of *subsymbolic* dynamics (i.e., non-conceptually). The attentional 1PP is conscious, but non-conceptual. What we call “attention” when using folk-psychological, everyday concepts, and when describing the mind from a third-person perspective, is a process by which the brain allocates computational resources to regions in which a deeper, more detailed form of processing is needed. In some cases, this is a saliency-driven, bottom-up process, in other cases involving “top-down attention,” it involves a kind of inner agency, an active process of selecting certain perceptual or cognitive contents for closer inspection and deeper processing. It is important to note, however, that it is one thing for a system to *have* the capacity for selective, top-down attentional control, and another for the same system to also *know* that it possesses this capacity. One centrally relevant theoretical idea is that the conscious experience of directing one's attention involves creating an internal model of an ongoing process of resource allocation, thereby establishing a self-representational kind of knowledge for the organism. The essence of this knowledge is that the organism is not only a bodily, but also an *epistemic* agent—a being that is currently attempting to expand its knowledge by actively directing its own capacities for information processing at the world and at its own states.

If this is correct, then it is clear why dream research is centrally relevant for the project of understanding how a “knowing self,” a *subject of experience* emerges. Dream researchers and empirically informed philosophers have collaborated to investigate those changes in the human self-model that are necessary in causally enabling the transition from non-lucid to lucid dreaming, have recently made progress in conceptually differentiating stages of lucidity as well as in isolating the neural correlates of this specific target property (Windt and Metzinger, 2007; Voss et al., 2009; Noreika et al., 2010; Dresler et al., 2012; Voss et al., 2013). Obviously, this kind of research possesses great relevance for the philosophy of mind, because it gives us a much deeper understanding of what we mean by concepts like “subjectivity” or “epistemic agency.”

Here is one example how dream researchers could make a contribution to the project of grounding the 1PP. The spatial correlation between eye-movements in the dream body and the physical body has served as the foundation for a number of ingenious studies homing in on the neural correlate of “lucidity” (i.e., the rare occurrence of realizing that one is dreaming during the dream state itself; see LaBerge et al., 1981; Voss et al., 2009, 2013; Dresler et al., 2012; for discussion see Metzinger, 2003a; Windt and Metzinger, 2007; Windt, 2014). In these experiments, lucid dreamers actively send signals to the experimenter via deliberate eye movements, which can be read off the EOG and are retrospectively confirmed by the dreamers themselves. This discovery is theoretically important, because it gives us a first candidate for a *grounding relation* connecting the conscious dream body with low-level physical dynamics (measurable neural activity controlling eye movements), perhaps linked through an unconscious body-model mediating the active exploration of visual space (Dement and Kleitman, 1957; Leclair-Visonneau et al., 2010). Phenomenal self-consciousness and self-related cognition can be grounded in different ways, including neurally realized simulations, bodily processes, and situated action (Barsalou, 2008: 619). Interestingly, here we find all three of them: Gaze control in the lucid dream state clearly is a form of virtually situated action, because it involves motor control plus attentional resource allocation in a virtual environment; it uses an internal simulation of the body-as-visually-attending (the self-model of the dream state, see Windt and Metzinger, 2007; Windt, 2014 for details); and it has dynamic, structure-preserving bodily correlates. The successful control of gaze direction in the lucid dream state is what anchors selective, high-level visual attention (including the conscious sense of “attentional agency”) in low-level, non-representational features of bodily movement. Here we have an example of a specific and functionally persistent grounding relation connecting the origin of the 1PP to unconscious processes, and in a situation where bodily action is almost entirely absent. It would be a valuable contribution to describe the necessary and sufficient conditions for this grounding relation to be realized in the dream state in a more precise way, and especially the temporal dynamics, the different stages though which it appears and disappears in the dream state. One might also interestingly connect this to the issue of describing the “minimal unit of identification” discussed in Part 1: Are there any relevant differences in bodiless dreams or asomatic OBEs? Perhaps bodiless dreams are the class of phenomenal states in which the relationship between MPS and the emergence of an EAM can be studied best.

### Example 2: cognitive phenomenology, mind wandering, and mental autonomy

“Cognitive phenomenology” is a label for a new subfield of research in philosophy of mind that focuses on the phenomenal character of occurrent non-sensory mental states like thoughts or wishes, on the distinct subjective quality that goes along with thinking (see Bayne and Montague, 2011 for a good overview). Some philosophers claim that there is a *proprietary, distinctive* and *individuative* phenomenology of higher cognitive processing that cannot be derived from sensory phenomenology, others deny this claim. Very obviously, research on the phenomenology of mentation in REM- and NREM-sleep, or on the characteristics of the thought process during lucid vs. non-lucid dreaming is directly relevant here (Box 2).

“Mind wandering” names a new field of research in psychology that has important, but unexplored connections to dream research and implications for cognitive phenomenology as well (Schooler et al., 2011; Metzinger, under review). Mind wandering takes place during perceptual decoupling, when spontaneously occurring mental events disengage attention from perception, leading to stimulus-independent trains of thoughts, for example during episodes of “daydreaming” or “zoning out” while reading (Smallwood and Schooler, 2006). In the absence of demanding external tasks, during routine activities or while resting we often lose the quality of attentional agency as defined above. Attentional focus is “hijacked” by representational processes not focused on the here and now any more, and importantly, we are often unaware of this very fact for extended periods of time. Mind wandering takes place during 30–50% of our waking life (Kane et al., 2007; Killingsworth and Gilbert, 2010; Schooler et al., 2011). Empirical findings show that we have the ability to take explicit note of ourselves as engaging in mind wandering, but only intermittently and only rarely. While mind wandering clearly is a recurring, marked loss of cognitive control and interferes with online sensory processing (for an excellent, concise overview of performance costs, see Mooneyham and Schooler, 2013, p. 12, Table 1) it may also possess an important functionality for creativity (Baird et al., 2012), attentional cycling in multiple-goal configurations, dishabituation, or autobiographical planning (Baird et al., 2011; Mooneyham and Schooler, 2013; Stawarczyk et al., 2013).

Conceptually, mind wandering and dreaming are both interesting to philosophers, because they involve a cyclically recurring decrease in mental autonomy that is not self-initiated and frequently unnoticed. Autonomy is the capacity for rational self-control, whereas the term “mental autonomy” (or “M-autonomy”; see Metzinger, under review; Box 2) refers to the specific ability to control one's own mental functions, like attention, episodic memory, planning, concept formation, rational deliberation, or decision making. NREM-sleep mentation and non-lucid dreaming clearly are also periods during which the functional property of M-autonomy is absent, although complex cognitive processes are taking place across all sleep stages (Nielsen, 2000; Fosse et al., 2001; Fox et al., 2013; Klinger, 2013; Wamsley, 2013; Windt, 2014) and can be sampled, for example using a serial awakening paradigm (Noreika et al., 2009; Siclari et al., 2013). Here, my thesis is that the recurring *loss* of mental autonomy is one major characteristic of our cognitive phenomenology, and that both research on dreaming and mind wandering have developed important research tools to investigate this hitherto neglected aspect further (like external probing, or systematic questions after sleep laboratory awakenings; see also Smallwood, 2013). It is empirically plausible to assume that a considerable part of our own cognitive phenomenology simply results from a frequent failure of executive control (McVay and Kane, 2009). I would claim that this actually is one of the most important functional and phenomenological characteristics of human self-consciousness, as a matter of fact, one of its most general, principal features: The almost constant presence of subpersonal and automatically generated mental activity (as generated by the default-mode brain network; Raichle et al., 2001; Buckner et al., 2008), in combination with a frequent inability of the executive-control system to shield primary-task performance off against interference from these subpersonal thought processes. If I am right, autonomous cognitive self-control is an exception, and not the rule (Metzinger, under review).

There are many other important connections between dreaming and waking mind wandering. First, the occurrence of dream lucidity and what researchers in mind wandering call “meta-awareness” (i.e., the “explicit knowledge of the current contents of thought,” see Schooler et al., 2011, p. 321) share many features, such as restoring mental autonomy. On the other hand, lucid lapses and mind-wandering lapses can both plausibly be interpreted as the disintegration of the EAM: The subject of experience loses the quality of epistemic agency. Second, dreaming and mind wandering may both share *positive* functionalities, such as the encoding of long-term memory (Christoff et al., 2011: 263p.), complex, preparatory motor planning and prospective autobiographical planning, or creative incubation. For example, Mooneyham and Schooler (2013: 15) hypothesize that “mind wandering may play a role in successful incubation (i.e., in coming up with novel solutions to previously presented problems when presented with them after the incubation period).” Third, there is considerable overlap between the neural correlates of mind wandering (Gruberger et al., 2011; Christoff, 2012) and those of the dream state (Solms, 2011), giving new support to the classical idea that there may be an uninterrupted continuum between controlled personal-level thought in wake states, mind wandering, and dreaming as an “intensified” version of mind wandering (Fox et al., 2013, figure 3; Wamsley, 2013). Fourth, in dream research there is the theoretical problem of “false lucidity”: There are reasons to believe that some subjects reporting lucid dreams were actually awake and successfully controlling the plot of their waking fantasies. In addition, there are different concepts of “lucidity” in academic research (Noreika et al., 2010; Voss et al., 2013), but do all subjects possess mastery of the *concept* of “lucidity”? If yes, what *is* their concept of lucidity? In mind wandering, the experience of *oneself* having actively regained meta-awareness (and thereby mental autonomy) could be an illusion of control over a mental event, which was really triggered by an unconscious process (Wegner, 2002; Schooler et al., 2011, Box 1). Although mostly neglected by philosophers^12^, both theoretical issues are directly relevant to the project of cognitive phenomenology. In particular, the specific “phenomenology of insight” going along with becoming lucid in a dream or with successfully catching oneself mind-wandering may prove to be of central importance: Is this phenomenology an epiphenomenal, *post-hoc* confabulation on the level of our conscious self-model, or does it possess a genuine epistemic status and a distinct causal role in mental self-regulation?

### Example 3: embodiment

For a quarter century now, the notion of “embodiment” has been central in philosophical discussions of the relationship between intelligence and its physical basis (see Robbins and Aydede, 2009; Shapiro, 2012, 2014). How do properties of the body constrain our model of the world? Does the body itself play a constitutive role in cognitive processing? What is the role of unconscious and conscious body representations for higher forms of intelligence and for the phenomenology of bodily self-consciousness? To give an example, in earlier publications, I have claimed that the dream state, which is accompanied by sleep paralysis, often is an example of explicit body-representation (we experience a dream body), but in the absence of active low-level embodiment, simply because the physical body is inert and fully paralyzed^13^. Dreamers, therefore, are not fully embodied agents (e.g., Metzinger, 2003a: 256, Metzinger, 2009). The “functional disembodiment hypothesis” (Windt, 2014) says that the dream state is characterized by a functional disconnection from the sleeping body such that real-body inputs do not enter into the dream and internally experienced dream movements are not enacted by the sleeping body. On this view, the phenomenal body is completely independent of the physical body. I also pointed out a single, highly interesting exception, namely the reliable directional correspondence between dream-eye movements and real-eye movements: There is at least one sense in which the phenomenal dream self is not completely disembodied in the functional sense (Metzinger, 2003a, p. 261; see the discussion of AA above).

The *generalized* version of the functional disembodiment claim, however, has now been refuted, as it can be shown that more than one minimal form of functional embodiment is preserved during REM-sleep dreams (cf. Windt, 2014, chapter 8). Real-body stimulation (e.g., sprays of water on the skin, Dement and Wolpert, 1958; electric stimulation, Koulack, 1969; blood-pressure-cuff stimulation on the leg, Nielsen, 1993; Sauvageau et al., 1993, 1998; Nielsen et al., 1995; vestibular stimulation, Hoff, 1929; Hoff and Plötzl, 1937; Leslie and Ogilvie, 1996) is frequently incorporated in dreams, and indeed it has been suggested that many typical dream themes—such as dreams of flying, falling, or being unable to move or flee from a pursuer—can be explained in terms of illusory own-body perception (Schönhammer, 2004, 2005), and the same may be true for sleep-paralysis nightmares during sleep onset (Cheyne et al., 1999; Cheyne, 2003, 2005). An early predecessor of this view of dreams as *weakly functionally embodied* states (Windt, 2014) is the *Leibreiztheorie*, promoted by 19th century researchers and extensively discussed and rejected by Freud (1899/2003, pp. 38–56). On the output side, there is also ample evidence for dream-enactment behavior in healthy subjects (e.g., Nielsen et al., 2009) and patients with RBD (REM-sleep behavior disorder; see Schenck, 2005, 2007; Leclair-Visonneau et al., 2010), as well as some evidence from lucid dreaming suggesting that dream movements are accompanied by measurable muscle twitches in the corresponding limbs and changes in heart and respiration rate (LaBerge et al., 1981, 1983; LaBerge and Dement, 1982; Fenwick et al., 1984; Schredl and Erlacher, 2008).

Are there any empirical examples of bodily experience or stable cognitive processing in dreams being created completely offline? Windt (2014, chapter 8) has investigated the issue and points out that, at the very least, it would be hard to see how there could be any empirical evidence for saying that such instances of functionally disembodied self-consciousness exist in dreams: state-of the-art studies investigating the sensory input blockade (Hobson et al., 2000), or, as Dang-Vu et al. more aptly call it, the “reduced sensitivity to external salient stimuli” (Dang-Vu et al., 2007, p. 1000) during REM sleep not only explicitly acknowledge that the degree of sensory incorporation is weakened rather than completely absent in REM sleep, but also use stimulus incorporation as an important means of *studying* the underlying mechanisms in the first place. This suggests that the most plausible and parsimonious explanation of bodily experience in dreams, as well as the most effective methods used for its study, will appeal to its real-bodily basis. But there are important conceptual issues to which dream researchers can make valuable contributions: The phenomenology of embodiment is grounded in unconscious body representation, which in turn “bottoms out” in microdynamical processes best described as non-representational, and which do not involve anything like explicit computation over discrete, symbolic states (Metzinger, 2014). But what exactly does “grounded cognition” (Barsalou, 2008) in the dream state really mean?

There are a number of relevant contact points between dream research and the burgeoning field of embodiment in philosophy and cognitive science. The two most important questions may be the following: Can the multiple cognitive deficits seen in the dream state be better understood if we analyze them as *deficits in embodiment*, bringing the whole armory of new conceptual tools developed in current philosophical discussions of embodiment (Shapiro, 2014), situated cognition (Robbins and Aydede, 2009), or active externalism (Clark and Chalmers, 1998; Menary, 2010) to bear on the problems at hand? Are there deeper systematic and/or functional reasons beyond the brain alone that lead to the multitude of mental impairments we see during dreaming? Second, if we aim at a comprehensive theory for the *phenomenology* of embodied selfhood, empirical constraints produced by dream research can be extremely fruitful: How determinate (or indeterminate) is oneiric body representation really (Windt, 2014)? What is the minimal unit of identification (see Part 1 above), and how can dream research inform conceptual work about the necessary and sufficient conditions for MPS (Blanke and Metzinger, 2009; see section Minimal Phenomenal Selfhood: The Original Hypothesis above)?

### Example 4: self-deception, epistemology and the argument from cognitive corruption

Could there be a radical form of philosophical skepticism, much more radical than the systematic process of doubt in Descartes methodological skepticism, which claims that even during the most critical, rational self-reflection it is possible to be fundamentally wrong about the reliability of one's own cognition? Could we enjoy the cognitive phenomenology of rationality, insight, or certainty while being fundamentally wrong about the epistemic status of our own mental states? Dream research, it seems, delivers a direct proof of concept. It demonstrates that unnoticed rationality deficits are possible at any point of our conscious lives. Jennifer Windt is the first author to systematically investigate this particularly promising contact point between philosophy and dream research, which has mostly been overlooked by professional epistemologists. She writes:

In a dream, one can have the impression of engaging in rational thought or remembering something about one's waking life and be completely wrong. The phenomenology of knowing, thinking, and remembering seems particularly vulnerable to this type of corruption in the dream state. This type of dream deception, then, is not so much deception about the nature of the dream world as deception about the reliability of one's current cognitive abilities. It is epistemologically troubling because it brings the threat of deception even closer to home: whereas Cartesian dream deception has us deceived about the perceptual world and our bodies, *deception from corrupted cognition* has us deceived about our minds. Consequently, we can never be sure of being truly rational, at any given moment. (Windt, 2014, section 10.2.2)

This point, however, is not only of great relevance to philosophical epistemology and the project of a “cognitive phenomenology” discussed above (in section Example 1: The Concept of a “First-Person Perspective”). It also bears on the methodology of dream research itself, because it calls into doubt the status of dream reports. In principle, subjects in sleep laboratories could suffer from massive memory losses or distortions, or confabulate about their own phenomenology without knowing (Rosen, 2013). In practice, however, when taking into account all our background knowledge, prior probabilities and considerations of simplicity, we are justified in a simple inference to the best explanation stating that dream reports, at least when gathered under certain ideal reporting conditions, will be veridical (Windt, 2013).

## Conclusion

In conclusion, we can now name a short, non-exhaustive list of desiderata for future research, focusing on new interdisciplinary targets connecting the philosophy of mind and empirical dream research:

- **Isolating the property of minimal phenomenal selfhood (MPS):** Much more precise data on bodiless dreams are urgently needed. I have hypothesized that the sense of selfhood seems to remain robust even in those asomatic OBEs and bodiless dreams in which there is neither an experience of motion in space nor goal-directed mental activity.
  ◦ Can this hypothesis be supported by new data?
  ◦ How can dream research inform conceptual work about the necessary and sufficient conditions for MPS?
- **Describing the phenomenal unit of identification (UI)**: It has been shown that existing empirical material on asomatic OBEs fails to distinguish between cases in which the only locus of identification is the *minimal* unit of an extensionless point in space and cases in which it is the *maximal* unit (as in parasomatic OBEs of the “pure consciousness” variant).
  ◦ Obviously, the same problem exists for bodiless dreams; new questionnaires should be designed to resolve this ambiguity.
- **Taxonomy**: There is now a new way to *categorize* dreams, namely, by their unit of identification (UI).
  ◦ How can questionnaires be optimized in order to reliably pick out the UI, turning it into a new dimension in scientific taxonomies of dreaming?
- **Isolating the epistemic agent model (EAM)**: How can dream researchers help to isolate the EAM in its purest form, and how can it contribute to describing the *transition* from MPS to EAM (e.g., in lucidity onset)?
- **Dissociating functional levels of the first-person perspective (1PP)**: Which specific contributions can dream research make to the project of grounding the 1PP?
- **1PP and EAM**: Is the “epistemicity” of the EAM a necessary condition for the occurrence of a robust form of phenomenal perspectivalness (1PP)?
  ◦ Is every conscious subject necessarily a (phenomenologically) *knowing* self, or should we treat the conjunction of “transparency” and “self-location” in itself as a sufficient condition?
- **Lucidity and EAM**: Is it really true that extended bodiless dreams mostly occur in lucid dreams?
  ◦ Here, the falsifiable hypothesis is that a stable EAM is necessary to extend such dreams beyond brief, snapshot-like episodes. More data are needed.
- **Phenomenology**: One empirical prediction the current proposal makes is that *transparent* spatiotemporal self-location is necessary and sufficient for bringing about a minimal, consciously experienced sense of selfhood.
  ◦ Do we know any cases of phenomenally *opaque* self-location, i.e., cases in which the location in a spatiotemporal frame of reference is *itself* consciously experienced as a form of representational content, as something that is not directly given, but as an internal construct? Is MPS instantiated in these cases?
  ◦ Dissociating self-location and self-identification: Are there *fully* lucid, phenomenally opaque, and bodiless dreams for which it is true that spatiotemporal self-location is itself experienced *as a form of (mis)representational content*, but in which the phenomenal dimension of self-identification is not given?
- **Embodiment:** The *depth* of embodiment during dreaming should be investigated in greater detail, because it sheds light on the different ways in which the body structures and anchors the phenomenal, representational, and behavioral spaces we navigate.
  ◦ Can the multiple cognitive deficits seen in the dream state be better understood if we analyze them as *functional deficits in embodiment*?
  ◦ How determinate (or indeterminate) is oneiric body representation?
- **Cognitive phenomenology**: How can dream research inform debates in philosophy of mind, for example by elucidating the hitherto neglected nature of the frequent, cyclical recurrence of loss of mental self-control?
- **Mind wandering**: What exactly is the relationship between mental self-control, the occurrence of dream lucidity and what researchers in mind wandering call “meta-awareness”?
  ◦ Can lucid lapses and mind-wandering lapses plausibly be interpreted as the disintegration of the EAM?
  ◦ Are there common *positive* functionalities connecting dreaming and mind wandering during wake states, such as the encoding of long-term memory, complex, preparatory motor planning, or creative incubation?
  ◦ False lucidity and the phenomenology of insight: In becoming lucid and in daytime mind wandering, is the experience of *oneself* having actively regained meta-awareness (and thereby mental autonomy) an illusion of control over a mental event that was really triggered by an unconscious process? Are there other common functionalities?
- **Epistemology**: How can dream research inform technical debates in philosophical epistemology, for instance though the “argument from cognitive corruption” sketched above?
  ◦ What *methodological* consequences do such debates have for dream research itself?

In conclusion, it has become clear that philosophers and dream researchers still have a lot to learn from each other, and that interdisciplinary cooperation should and must be further developed. In order to directly support this goal, the current contribution has tried to isolate a set of particularly promising research targets and described specific interdisciplinary contact points. Viewed from the systematic, metatheoretical perspective of philosophy of mind, the MPS-problem may be the most relevant and innovative entry point, because it helps to *conceptually unify* the problem landscape in a new way. Dreams and phenomenal states are *subjective* states, and in order to understand the constitutive conditions determining the gradual emergence of a subject of experience, we need to understand the fundamental phenomenology of transparent self-identification that is common to all levels of conscious subjectivity.

### Conflict of interest statement

The Author, Editor and Chief Editor declare that while the author Thomas Metzinger and the editor J. Windt are currently employed by the same institution (Johannes Gutenberg-Universität, Mainz, Germany) there has been no conflict of interest during the review and handling of this manuscript. The author declares that the research was conducted in the absence of any commercial or financial relationships that could be construed as a potential conflict of interest.

## Footnotes

^2^“Transparency” is a property of conscious representations, namely, that they are not experienced *as* representations. Therefore, the subject of experience feels as if being in direct and immediate contact with their content. Transparent conscious representations create the phenomenology of naïve realism (see next footnote and the references given there). An opaque phenomenal representation is one that is experienced *as* a representation, for example in pseudo-hallucinations or lucid dreams. There exists a graded spectrum between transparency and opacity, determining the variable phenomenology of “mind-independence” or “realness.” Unconscious representations are neither transparent nor opaque.

^3^The phenomenology of “realness” can be analyzed as the *phenomenal transparency* of certain conscious representations, i.e. as the fact that the system has no introspective access to non-intentional properties of its own representations, that it is necessarily unaware of the construction process (see Metzinger, 2003a: 3.2.7 and Metzinger, 2003b: 354pp for details). If we move from the representationalist to the functionalist level of analysis, we may interpret experiential “realness” as the Bayes-optimality or dynamical stability of the generative models employed. On this level, my claim would be that the origin of the 1PP (the minimal phenomenal self) is not tied to a specific form of *content*, but to the region of maximal invariance, that is, to the most robust, dynamically stable, and Bayes-optimal region in the overall model of reality. There is a “gradient of realness,” and the most real (or “phenomenally transparent”) partition of the conscious model of the world is the aspect which minimizes prediction error most reliably; cf. Limanowski and Blankenburg (2013).

^4^An even stronger 1PP emerges if a system represents itself as an *epistemic agent*, that is, as a system that actively seeks to expand or improve its knowledge about the world (Box 1). Two major ways in which this can be done is, first, by optimizing the precision of its perceptual model of the world via the selective control of attentional mechanisms and active sampling (“attentional agency”, AA, see section Example 1: The Concept of a “first-person perspective”), or second, by forming new concepts and abstract representations of reality (“cognitive agency”). A strong 1PP occurs when the model of the organism as a whole, given through MPS, is represented as being directed at an object component (including, potentially, the body itself). A cognitive 1PP appears when a system possesses an abstract concept of itself as a subject of experience, and is able to apply this concept to itself. See Metzinger, 2003a, 2006, 2007 for details.

^5^I have called this the “embedding principle.” For empirical examples demonstrating how both aspects can be functionally dissociated, suspending the embedding principle, see De Ridder et al., 2007; Ehrsson, 2007; Lenggenhager et al., 2007.

^6^Heterophenomenological constraints are third-person constraints for a scientific theory of subjective experience, exclusively based on the *reports* people give about their own experience, and not on some dubious form of direct access to “the experience itself.” Daniel Dennett (2003, p. 20) claims “The total set of details of heterophenomenology, plus all the data we can gather about concurrent events in the brains of subjects and in the surrounding environment, comprise the total data set for a theory of human consciousness. It leaves out no objective phenomena and no subjective phenomena of consciousness.”

^7^As Alvarado (1997, p. 16) remarks, little systematic work has been conducted on the phenomenology of the experience (see also Alvarado, 1986, 2000, p. 186). In autoscopy, the object-component of the first-person perspective—what the subject is phenomenally *directed* at—is formed by a self-model which is not a *subject*-model, it is not a representation of a knowing self. You see your own body, and you recognize it as your own, but presently it is not the body *as subject*, the body as the locus of knowledge and of lived, conscious experience –there are two bodies, but only one EAM. The phenomenal “ownership” for the physical body is based on instant recognition only, but not on identification. Only one of the currently active self-models functions as the “locus of identification,” namely the representation of an epistemic agent, the currently knowing self. It is also interesting to note how OBEs, phenomenologically, are *not* states of full disembodiment. On the contrary, there always seems to be a spatially located phenomenal self, even if its embodiment is reduced to a purely spatial point of epistemic agency. Perhaps, it would therefore be better to speak of mislocation or “misembodiment”

^8^For an introduction and references see Fasching (2008).

^9^A further important conceptual issue is the possibility of *phenomenal indeterminacy*. This means that in some cases there may not even have been a “phenomenological fact of the matter” concerning the object of identification. In her 1982 monograph, Susan Blackmore discusses findings of Green, Poynton, Rogo and the *Society for Psychical Research* and points out an interesting possibility: “This indicates that for many OBEers the question of whether or not there is another body is so uninteresting that they say nothing about it, even in accounts several pages long. Perhaps this is part of the reason for varied results. Even if you force people to answer the question they may not really know whether they had another body or not.” (cf. p. 66) This supports what Windt (2014, chapter 7) says about the phenomenal indeterminacy of bodily experience, namely that phenomenal disembodiment is probably rare, but that phenomenal indeterminacy in dreams might be quite widespread, though typically unreported. However, unlike Blackmore, Windt defends the additional claim that this is not just a factor of not *knowing* whether or not one had a body, but of there being no fact of the matter in experience itself. Interestingly, this is also the most complete way of lacking a body, because these are not even cases of feeling the absence of the body as in “pure self-location”, but of the body dropping out of experience altogether.

^10^For the purposes of this paper I will ignore the problem of *temporal* self-location and the issue of mental self-location in a temporal frame of reference. Clearly, there is a minimal form of temporal content as well, and it is an important source of invariance. See (Metzinger, 2003a), sections 3.2.2 and 6.2.2, and (Metzinger, under review).

^11^We can now characterize lucid dreams as those in which the brain has already succeeded in establishing a stable EAM, whereas non-lucid dreams appear more as an ongoing, and sometimes desperate, attempt to *consolidate* enduring epistemic relationships to the dream world, of gaining control over attention and one's own cognitive processing, the same point applies for mind wandering, see Metzinger (under review). The non-lucid dreamer experiences epistemic agency mostly as a search for stability, or an ongoing, constantly perturbed attempt to understand the situation, whereas the lucid dreamer is capable of goal-directed mental behavior, in thinking, remembering, and focusing attention on specific target objects. A helpful and more detailed discussion of “cogitative” and “doxastic” indeterminacy as characterizing cases where phenomenal epistemicity is lost can be found in (Windt, 2014) (Chapters 9 and 10).

^12^Notable exceptions are Jean-Paul Sartre (1940), Colin McGinn (2005, 2009) or Jonathan Ichikawa (2008; for a critical philosophical evaluation, see (Windt, 2014), chapter 6). Sartre, McGinn, and Ichikawa all assume that we control our daydreams, and try to show that the same is true for nocturnal dreams – but based on empirical evidence from research on mind wandering (see section 4.2), this claim is even empirically implausible with respect to daydreaming itself.,

^13^This section draws on a discussion also presented in (Metzinger, 2014).
